# Adolescent and Parent Experience of Care at a Family-Based Treatment Service for Eating Disorders

**DOI:** 10.3389/fpsyt.2020.00310

**Published:** 2020-04-21

**Authors:** Elizabeth K. Hughes, Suzannah Poker, Amy Bortz, Michele Yeo, Michelle Telfer, Susan M. Sawyer

**Affiliations:** ^1^Department of Pediatrics, University of Melbourne, Melbourne, VIC, Australia; ^2^Murdoch Children’s Research Institute, Melbourne, VIC, Australia; ^3^School of Psychological Sciences, University of Melbourne, Melbourne, VIC, Australia; ^4^Melbourne Medical School, University of Melbourne, Melbourne, VIC, Australia; ^5^Department of Adolescent Medicine, Royal Children’s Hospital, Melbourne, VIC, Australia

**Keywords:** anorexia nervosa, adolescents, family therapy, consumer research, patient and family-centered care

## Abstract

**Objective:**

Incorporating consumer perspectives is an important but often overlooked opportunity to optimize treatment engagement and outcomes for adolescents with eating disorders. This study explored the experience of care of adolescents and their parents at a multidisciplinary specialist eating disorders service providing family-based treatment (FBT) as first-line treatment.

**Method:**

Eighty-five adolescents and 145 parents who completed FBT at the service between 2013 and 2015 were surveyed in 2017 about their experience of care. A study-designed survey asked respondents to rate on Likert scales their experience of service access, intake assessment, education, support, interactions with the treatment team, recovery, and the discharge process. Open-ended comments on helpful and unhelpful aspects of the service provided further context on the ratings.

**Results:**

Overall families were very positive about their experience, particularly in regard to assessment, education, interactions with the team, and achieving physical health. Although parents tended to be more satisfied, adolescents also held the service in high regard. Some areas were identified that could be improved, including treatment delays, carer support, therapeutic alliance, and preparation for discharge.

**Conclusions:**

Surveying families about their experience of care provides an important opportunity to identify service strengths as well as services gaps. The results indicated several areas that specialist eating disorder services could focus on to ensure that the services provided, including FBT, fully meet the needs of families and optimize adolescents’ treatment experiences.

## Introduction

The importance of consumer perspectives for effective evidence-based practice (1, 2) has received increasing recognition in relation to optimizing treatment of eating disorders (3). For adolescents with anorexia nervosa (AN), current evidence supports family-based treatment [FBT (4)] preferably within a specialist multidisciplinary service (5–7). Despite its strong evidence base (8), FBT only achieves full remission in approximately one third of adolescents, although partial remission rates are higher (9–12). Treatment engagement can be especially challenging for families and clinicians given the ego-syntonic nature of AN (13) and the intense demands of FBT for the family. Therefore, exploring adolescents’ and parents’ experience of care within a specialist FBT service may identify important opportunities to optimize treatment engagement and outcomes (3).

Few studies have examined the experience of adolescents with AN, particularly in relation to FBT. A recent qualitative meta-synthesis identified 15 studies exploring patients’ experience of family therapies for AN (14). However, only nine of these explored adolescents’ experience of which only two were of adolescents who had received FBT (15, 16). Nonetheless, the review identified several key aspects of treatment viewed as helpful by patients including parental control of eating, externalization of the illness, reduced criticism, and improved family relationships. In contrast, aspects of treatment perceived as unhelpful included lack of attention to underlying issues, failure to address some family issues, and an unmet need for individual therapy. Similar perspectives regarding the helpfulness of aspects of FBT have been reported by parents (16) and found in studies of parents’ and patients’ experience of variants of FBT, including separated and inpatient delivered FBT (17–19). Moreover, when both parent and patient perspectives are included, parents are generally more positive about FBT than their children (16–19).

Few studies have examined the experience of adolescents with AN, particularly in relation to FBT. A recent qualitative meta-synthesis identified 15 studies exploring patients’ experience of family therapies for AN (14). However, only nine of these explored adolescents’ experience of which only two were of adolescents who had received FBT (15, 16). Nonetheless, the review identified several key aspects of treatment viewed as helpful by patients including parental control of eating, externalization of the illness, reduced criticism, and improved family relationships. In contrast, aspects of treatment perceived as unhelpful included lack of attention to underlying issues, failure to address some family issues, and an unmet need for individual therapy. Similar perspectives regarding the helpfulness of aspects of FBT have been reported by parents (16) and found in studies of parents’ and patients’ experience of variants of FBT, including separated and inpatient delivered FBT (17–19). Moreover, when both parent and patient perspectives are included, parents are generally more positive about FBT than their children (16–19).

Although informative, consumer studies of FBT to date have been small (i.e., maximum 46 adolescents and 66 parents), and have largely focused on the helpfulness of aspects of FBT rather than the broader service setting. A handful of larger community-based studies have explored consumer perspectives on eating disorder treatment services. These have typically involved adults with a variety of eating disorder diagnoses and asked them to reflect on what they value in services. In one survey of current and former patients with eating disorders (n = 304) in The Netherlands (20, 21), the most highly valued aspects focused predominantly on the personal qualities of the therapist and the therapeutic relationship (e.g., trust in the therapist, being taken seriously, being able to talk about feelings, being respected). Therapists surveyed in this study (n = 73) also valued these aspects but tended to place greater importance on the content of therapy than did patients (e.g., learning to eat normally, recovery of weight, improving body image). A similar study (22, 23) surveyed patients (n = 196), carers (n = 79), and clinicians (n = 136) in the USA and UK. In that survey, patients rated the personal and professional qualities of the staff as the most important features of a high quality service, followed by the provision of psychological interventions. For carers and clinicians, professional qualities of the staff were also of high importance; however, support for carers and service availability and access were also rated as highly important.

This pattern of findings was confirmed in a review of 23 qualitative studies of the consumer experience of eating disorder treatments (24), with empathic and supportive relationships and psychological interventions viewed as the most helpful. This review also found that medical interventions and interventions focusing on food and weight were viewed more negatively. Such findings are not surprising given the ego-syntonic nature of AN and the distress that changing behaviors related to food and weight may provoke. Indeed, Swain-Campbell and colleagues (25) found that among 120 young adults treated at a specialist eating disorder service in New Zealand, many disliked components of treatment typically deemed essential by clinicians, such as gaining weight and loss of compensatory behaviors. Yet as Bell (24) pointed out, despite the difficulties patients experience with these interventions, many may well understand their necessity.

In sum, only a number of small studies have explored consumers’ experience of FBT which have largely focused on the perceived helpfulness of specific aspects of this treatment. Conversely, larger studies of broader treatment settings have been conducted with adults with a range of diagnoses who have received a variety of treatments. What is lacking are larger studies that provide an understanding of the overall service experience of adolescents and parents who have received FBT at a multidisciplinary specialist service, the currently preferred treatment modality for adolescents with AN (5–7). This study therefore explored the experience of care of adolescents and parents with regard to the multidisciplinary specialist service setting in which they engaged in FBT (c.f. components of FBT). By soliciting formal feedback from adolescents and parents on their experience of care, the study aimed to identify aspects of service provision that could be improved, or fortified, with the ultimate objective of understanding how to promote greater engagement and more positive outcomes for patients and their families.

## Materials and Methods

### Setting

The study took place at a multidisciplinary specialist eating disorders program at a tertiary pediatric hospital in Australia. The program provides treatment for restrictive eating disorders and includes staff from pediatrics, psychiatry, nursing, dietetics, social work, and psychology. Following referral to the program, all families attend an intake assessment. This includes clinical evaluations with the adolescent by a pediatrician, psychiatrist, dietitian, and nurse. Parents complete an evaluation with an FBT therapist covering development history, family life, and the onset of eating disorder symptoms. Both the adolescent and their parents also complete a set of standardized interviews and questionnaires. The intake assessment is typically completed in the outpatient clinic over a single day, and concludes with the team discussing the diagnosis and treatment plan with the adolescent and their parents. Adolescents referred to the program via admission to the inpatient ward (e.g., following presentation to the emergency department) complete the intake assessment over several days during the admission.

Outpatient treatment involves FBT delivered by mental health clinicians alongside medical monitoring by pediatricians and nurses and, if needed due to the acuity of mental health comorbidities, consultations with a psychiatrist. A standard course of FBT is 18 sessions over 6 months, with extended treatment provided for complex cases. Following FBT, adolescents continue to be monitored by their pediatrician for as long as necessary, or until transfer to adult services at 18-19 years of age. During the period of this study, FBT was the only outpatient treatment provided. If further mental health care is required beyond the initial course of FBT, it takes place in the community. During treatment, adolescents can be admitted to the inpatient adolescent medicine ward if required; for example, if they become medically unstable (e.g., due to bradycardia).

### Participants and Procedure

The study was a retrospective cross-sectional survey undertaken in 2017. Participants were adolescents and their parents who received FBT at the service between 2013 and 2015. The period was selected to ensure adolescents had completed FBT and that there were sufficient participants for meaningful analysis. Parents could include mothers, fathers, step-parents, and grandparents if they were a primary caregiver involved in FBT. Both parents were eligible to participate in the survey if they had been involved in FBT, including separated families. The term “parents” is used given this represents the vast majority of participants. Participants were excluded if no current contact details were available (n = 11), they had previously indicated that they did not want to be contacted regarding research (n = 4), they could not read or speak English at a level that would allow them to complete the survey (n = 3), or the adolescent was still receiving FBT with the program (n = 5) or was under 12 years old (n = 1).

A total of 175 eligible families were identified. Each family was sent a study information statement by the head of the clinical department, and given a 2-week opportunity to opt out of participating. After this time, a researcher attempted to contact parents by phone. Once contacted, parents who agreed to participate were emailed a link to complete a survey online (88%) or posted a hardcopy survey (12%), depending on their preference. The adolescent survey was sent *via* the parent, or directly to the adolescent if the parent was willing to provide their child’s contact details. Often only the mother was able to be contacted by phone as this was the only number available in the adolescents’ record. In these cases, the mother had to be willing to supply the father’s contact information or pass on the survey to the father. If surveys were not completed after 1 week, a reminder email was sent. Reminder phone calls were made after 2 weeks, 4 weeks, and 6–10 weeks if the survey had not been completed.

The study was approved by the institutional human research ethics committee. Participant consent was implied by completion of the survey and parent/guardian consent was implied by parents providing the contact details of their child and/or passing on the survey to their child.

### Measures

A study-specific survey was designed which sought participants’ experience of the range of elements of the specialist program. This included access to the service (including delays), experience of the intake assessment, provision of education and information, support for parents during treatment, support for the adolescent during treatment, interactions with the treatment team (including communication and expertise), perceptions of recovery, and experience of the discharge process. The questions utilized five-point Likert Scale responses ranging from Completely Disagree to Completely Agree, or Completely Unsatisfied to Completely Satisfied. At the end of the survey, three open-ended questions invited participants to comment on what they found helpful about the program, unhelpful about the program, and any specific suggestions they had about what could be improved. The parent survey (120 items) and adolescent survey (94 items) were nearly identical apart from wording appropriate to the respondent’s role and some additional items for the parent (e.g., referral process, parent-specific aspects of FBT). Demographic and clinical information about the adolescent were also collected from existing records including sex, age, diagnosis, and weight at presentation.

### Analysis

The data were analyzed using SPSS version 24. For ease of interpretation, the five-point Likert scales were collapsed into three categories: Disagree (*Mostly Disagree* and *Completely Disagree*), Neutral, (*Neither Agree or Disagree*), and Agree (*Completely Agree* and *Mostly Agree*), or Unsatisfied (*Very Unsatisfied* and *Unsatisfied*), Neutral (*Neither Satisfied nor Unsatisfied*), and Satisfied (*Very Satisfied* and *Satisfied*). Missing data were under 5% across the survey items; no missing value replacement was undertaken. Descriptive statistics were calculated for each item. Answers to the open-ended questions were coded into domains covered by the quantitative rating scales. Representative quotes were then selected to demonstrate the quantitative findings for each domain explored in the survey.

## Results

### Sample Characteristics

Among the 175 eligible families (175 adolescents, 172 mothers, 153 fathers), 14 adolescents, 13 mothers, and 18 fathers and were unable to be contacted. A further 40 adolescents, 27 mothers, and 29 fathers declined participation. At the first phone contact, 50 mothers asked that the survey be sent only to them, and not to the father, despite the researcher requesting that both parents complete separate surveys. A total of 121 adolescents, 132 mothers, and 56 fathers were sent surveys. Ultimately, 85 adolescents (49%), 106 mothers (61%), and 39 fathers (22%) completed the survey. Thus, 120 families (69%) had at least one member participate, and 112 adolescents (64%) had at least one parent complete the survey.

Of the 85 adolescent participants, 80 (94%) were female, with a mean age of 18.4 years (SD =1.8; range 13.1–22.0) at the time of the survey. At presentation, adolescents had a mean age of 15.5 years (SD = 1.7; range 9.4–18.0), mean %mBMI of 86.8 (SD = 13.5; range 13.53–135.8), and were diagnosed with AN (n = 50; 59%), avoidant/restrictive food intake disorder (ARFID; n = 1; 1%), or other specified/unspecified eating or feeding disorder (n = 34; 40%; primarily atypical AN). At the time of the survey, 29 (34%) reported that they were currently engaged in regular treatment for an eating disorder.

The 145 parent participants comprised 104 mothers, 1 step-mother, 1 grandmother, 38 fathers, and 1 step-father. Given the smaller number of male caregiver participants, responses from all parents were combined for analysis. Most parents were born in Australia (79%) and spoke English as their main language (94%). At the time of the survey, 58 (40%) reported that their child was currently engaged in regular treatment for an eating disorder.

### Delays in Treatment Access

Around one-third of parents reported that they experienced a delay in being referred to or seen at the service from the time they became concerned, with 19 (13%) reporting a major delay, 29 (20%) reporting a minor delay, and 95 (66%) reporting no delay. A follow-up question prompted parents to describe the reason for any delay. The most common reasons given were that the primary healthcare provider (e.g., general practitioner) did not recognize or act on the eating disorder symptoms (n = 17), that treatment was sought from other professionals such as dietitians or psychologists before referral to the eating disorders program (n = 12), or there was a delay in getting an appointment with the program (n = 17).

*“We saw a GP, counsellor, psychologist, dietician and eventually a heart specialist for about 6 months before anyone even mentioned eating disorders. We only ended up at [the hospital] by accident due to an allergic reaction. Staff there [who were] experienced in eating disorders admitted her immediately.”*—Parent

### Experience of the Intake Assessment

Overall satisfaction with the intake assessment was very high for parents (96%) and somewhat lower for adolescents (72%), as can be seen in **Table 1**. Among parents, almost all felt they had an opportunity to talk about their concerns about their child (95%), that it was helpful to meet with a variety of professionals (97%) and that, at the end of the assessment, they were given clear information about whether their child had an eating disorder (96%) and understood how unwell their child was and why treatment was needed (96%). Somewhat fewer parents felt that other causes for their child’s eating or weight problems were considered (74%) or that additional physical or mental health problems their child were considered (74%) during the intake assessment. This was also the case for adolescents, among whom just 50% and 59% felt that other causes and problems were considered during the intake assessment, respectively. Of interest, only 55% of adolescents expressed that they felt safe and comfortable to talk about their problems at the intake assessment. Despite this, 84% of adolescents felt that, at the end of the assessment, they were given clear information about whether they had an eating disorder and 79% understood how unwell they were and why treatment was needed.

*“She was in a steep decline and I was completely lost and overwhelmed by the beast that is an eating disorder. I began to feel an inkling of reassurance and even hope from the moment we had our first appointment with the team.”*—Parent

*“I found the intake to be very overwhelming and scary because it was all in such a quick succession”*—Adolescent

**Table 1:** Experience of the intake assessment.

	Parents (n = 145)			Adolescents (n = 85)		
	n (%)	n (%)	n (%)	n (%)	n (%)	n (%)
	Disagree	Neutral	Agree	Disagree	Neutral	Agree
***Thinking about your intake assessment, how much do you agree with each of the following?***						
1. I was given an opportunity to talk about my concerns about my child (parents only)	4 (2.9)	3 (2.1)	133 (95.0)	–	–	–
2. I felt comfortable and safe to talk about my problems (adolescent only)	–	–	–	15 (17.9)	23 (27.4)	46 (54.8)
3. I was given an opportunity to talk about our family situation	5 (3.6)	8 (5.8)	126 (90.6)	9 (10.7)	13 (15.5)	62 (73.8)
4. It was helpful to have assessments completed by a variety of professionals (e.g., medical doctor, psychiatrist, dietitian, family therapist)	2 (1.4)	2 (1.4)	135 (97.1)	8 (9.5)	19 (22.6)	57 (67.9)
5. Other causes for my [*my child’s*] eating or weight problems were considered	15 (10.7)	22 (15.7)	103 (73.6)	21 (25.0)	21 (25.0)	42 (50.0)
6. Any additional problems I [*my child*] had were considered (e.g., other physical or mental health problems)	16 (11.4)	21 (15.0)	103 (73.6)	15 (18.1)	19 (22.9)	49 (59.0)
7. At the end of assessment, I was given clear information about whether or not I [*my child*] had an eating disorder	3 (2.2)	2 (1.4)	134 (96.4)	4 (4.8)	9 (10.8)	70 (84.3)
8. I was given a clear treatment plan	4 (2.9)	7 (5.0)	128 (92.1)	10 (12.2)	10 (12.2)	62 (75.6)
9. At the end of the assessment I understood how unwell I [*my child*] was and why treatment was needed	2 (1.4)	3 (2.2)	134 (96.4)	5 (6.1)	12 (14.6)	65 (79.3)
	**Unsatisfied**	**Neutral**	**Satisfied**	**Unsatisfied**	**Neutral**	**Satisfied**
***Overall, how satisfied were you with the intake assessment?***	2 (1.5)	3 (2.2)	131 (96.3)	7 (8.5)	16 (19.5)	59 (72.0)

### Education and Information

Parents and adolescents were asked about the amount of information they received about eating disorders and related issues during their time at the service. As can be seen in **Table 2**, most parents felt they received enough information about eating disorders (87%), how eating disorders affect physical health (84%) and mental health (77%), and where to find more information (81%). However, 31% of parents would have liked more information about evidence for treatment and 29% would have liked more information about medications. While most adolescents also felt they received enough information about what eating disorders are (80%) and how they affect physical health (73%) and mental health (68%), up to half wanted more information on prognosis (44%), evidence for treatment (48%), and medications (51%).

*“I found [the therapist] to be most helpful in providing us with the right information and knowledge needed to guide us in [our daughter]’s treatment and recovery.”*—Parent

*“They gave me the right information and guidance towards overcoming my eating disorder.”*—Adolescent

**Table 2:** Education and parent support during treatment.^a^

	Parents (n = 145)			Adolescents (n = 85)		
	n (%)	n (%)	n (%)	n (%)	n (%)	n (%)
	More	Enough	Too Much	More	Enough	Too Much
***During your time with the program, did you receive enough information about…?***						
1. What eating disorders are	15 (10.6)	122 (86.5)	4 (2.8)	10 (12.5)	64 (80.0)	6 (7.5)
2. The effect of eating disorders on physical health	18 (12.9)	117 (83.6)	5 (3.6)	13 (16. 3)	58 (72.5)	9 (11.3)
3. The effect of eating disorders on mental health	29 (20.4)	109 (76.8)	4 (2.8)	15 (18.5)	55 (67.9)	11 (13.6)
4. Prognosis (e.g., likelihood of recovery, time to recovery)	32 (22.9)	104 (74.3)	4 (2.9)	36 (44.4)	39 (48.1)	6 (7.4)
5. Evidence for treatment (e.g., findings from research studies showing the effectiveness of treatment)	43 (30.5)	94 (66.7)	4 (2.8)	38 (47.5)	37 (46.3)	5 (6.3)
6. Medications (e.g., effectiveness, side-effects)	34 (29.3)	78 (67.2)	4 (3.4)	37 (51.4)	31 (43.1)	4 (5.6)
***During your time with the program, did you receive enough guidance about…?***						
1. How to support your child at mealtimes	30 (21.3)	106 (75.2)	5 (3.5)	–	–	–
2. How to support your child not to vomit after eating	14 (17.1)	65 (79.3)	3 (3.7)	–	–	–
3. How to support your child not to exercise	25 (20.8)	92 (76.7)	3 (2.5)	–	–	–
4. How to help your child cope with weight gain	59 (44.4)	70 (52.6)	4 (3.0)	–	–	–
5. How to help your child when they were distressed	63 (45.0)	74 (52.9)	3 (2.1)	–	–	–
6. How to cope with your own distress about your child’s illness	73 (51.8)	65 (46.1)	3 (2.1)	–	–	–

a Response scale was More = I would have liked more information/guidance, Enough = I received enough information/guidance, Too Much = I received too much information/guidance.

### Support for Parents During Treatment

Parents were asked about the amount of guidance they received in several areas. As shown in **Table 2**, around three-quarters of parents reported that they received enough guidance in key areas related to FBT including how to support their child at mealtimes (75%), how to support their child not to vomit after eating (79%), and how to support their child not to exercise (77%). However, nearly half of the parents would have liked more guidance around supporting their child cope with weight gain (44%) and distress (45%) and more guidance on how to cope with their own distress about their child’s illness (51%).

*“I found the program helpful with having all doctors and other health care professionals around to support all of us and always give us advice on how to cope with the stressful times that we were going through.”*—Parent

*“We needed more support as parents. Our own mental health deteriorated as we put all our energy into getting our daughter well.”*—Parent

### Support for Adolescents During Treatment

Most parents were satisfied that the team regularly monitored their child’s physical health (98%), mental health (90%), and risk of self-harm/suicide (88%), and felt that their concerns for their child’s wellbeing and safety were responded to appropriately (90%), as shown in **Table 3**. Similarly, most adolescents reported that the team regularly monitored their physical health (93%) and mental health (82%); however, fewer adolescents agreed with the statement “*the team responded appropriately when I was feeling distressed*” (65%). The item regarding monitoring of safety was not asked of adolescents.

*“I wish there was more emphasis on dealing with the mental health side (DBT and CBT) but I do know that when a child is severely malnourished it is difficult for them to think clearly.”*—Parent

*“I felt like they weren’t taking my anxiety and depression into consideration and only considered the idea that anorexia caused the mental issues when in fact it was the other way around and when we tried to explain that, they weren’t listening.”*—Adolescent

**Table 3:** Support for the adolescent during treatment.

	Parent (n = 145)			Adolescent (n = 85)		
	n (%)	n (%)	n (%)	n (%)	n (%)	n (%)
	Disagree	Neutral	Agree	Disagree	Neutral	Agree
***How much do you agree with each of the following?*** ***The Program…***						
1. Regularly monitored my [*my child’s*] physical health (e.g., heart rate, blood pressure)	1 (0.7)	2 (1.4)	138 (97.9)	1 (1.2)	5 (6.2)	75 (92.6)
2. Regularly monitored my [*my child’s*] mental health (e.g., mood, anxiety)	6 (4.3)	8 (5.8)	124 (89.9)	8 (9.8)	7 (8.5)	67 (81.7)
3. Regularly monitored my child’s risk for self-harm or suicide (parent only)	4 (3.3)	11 (9.2)	105 (87.5)	–	–	–
4. Responded appropriately when I had concerns about my child’s wellbeing or safety (parent only)	5 (3.7)	8 (5.9)	123 (90.4)	–	–	–
5. Responded appropriately when I was feeling distressed (adolescent only)	–	–	–	10 (12.3)	18 (22.2)	53 (65.4)

### Interactions With the Treatment Team

Parents were very positive regarding the team’s expertise, communication, and interactions, with 89–99% of parents agreeing with the statements related to these areas, as shown in **Table 4**. Most adolescents also agreed that the team was knowledgeable and experienced with respect to eating disorders (both 94%) and that the team treated them with respect and courtesy (88%). However, fewer adolescents agreed that the team communicated well with them (70%), that they had confidence in the team’s ability to help them (67%), and that their concerns were listened to (64%) and acted on appropriately (64%). Of interest, 96% of parents did not feel the team blamed them for their child’s illness, and 74% of adolescents did not feel the team blamed them for their illness.

**Table 4:** Interactions with the team and overall satisfaction.

	Parents (n = 145)			Adolescents (n = 85)		
	n (%)	n (%)	n (%)	n (%)	n (%)	n (%)
	Disagree	Neutral	Agree	Disagree	Neutral	Agree
***How much do you agree with each of the following?*** ***Our treatment team…***						
1. …was knowledgeable about eating disorders	1 (0.7)	1 (0.7)	138 (98.6)	2 (2.4)	3 (3.7)	77 (93.9)
2. …was experienced with eating disorders	1 (0.7)	2 (1.4)	137 (97.9)	0 (0.0)	5 (6.1)	77 (93.9)
3. …communicated well with each other	3 (2.1)	5 (3.6)	132 (94.3)	6 (7.4)	6 (7.4)	69 (85.2)
4. …communicated well with me	4 (2.9)	8 (5.7)	128 (91.4)	11 (13.4)	14 (17.1)	57 (69.5)
5. …communicated well with my parents (adolescent only)	–	–	–	9 (11.0)	8 (9.8)	65 (79.3)
6. …gave us consistent information (i.e., they were in agreement with each other, didn’t give conflicting advice)	3 (2.2)	8 (5.8)	128 (92.1)	10 (12.3)	10 (12.3)	61 (75.3)
***How much do you agree with each of the following in regard to your treatment team?***						
1. I was treated with respect and courtesy	3 (2.2)	1 (0.7)	133 (97.1)	6 (7.3)	4 (4.9)	72 (87.8)
2. I had confidence in the team’s ability to help me [*my child*]	3 (2.2)	5 (3.6)	131 (94.2)	11 (13.4)	16 (19. 5)	55 (67.1)
3. The team had confidence in my ability to help my child (parent)/The team had confidence that I could get better (adolescent)	3 (2.2)	13 (9.4)	123 (88.5)	8 (9.8)	10 (12.2)	64 (78.0)
4. They didn’t blame me for my child’s illness (parent)/They didn’t blame me for my illness (adolescent)	4 (2.9)	1 (0.7)	135 (96.4)	9 (11.0)	12 (14.6)	61 (74.4)
***How would you rate the overall care and treatment you received from the program clinicians…?***	**Unsatisfied**	**Neutral**	**Satisfied**	**Unsatisfied**	**Neutral**	**Satisfied**
1. Your family-based treatment therapist	15 (11.3)	7 (5.3)	111 (83.5)	21 (26.6)	13 (16.5)	45 (57.0)
2. Your pediatrician/medical doctor	6 (4.5)	4 (3.0)	122 (92.4)	6 (7.3)	5 (6.1)	71 (86.6)
3. Psychiatrist	8 (7.5)	12 (18.7)	87 (81.3)	8 (12.9)	7 (11.3)	47 (75.8)
***Overall, how satisfied were you with the program as a whole?***	8 (6.0)	7 (5.3)	118 (88.7)	9 (11.0)	14 (17.1)	59 (72.0)

Most parents were satisfied with the care and treatment they received from their FBT therapist (84%), pediatrician (92%), and psychiatrist (81%). Similarly, most adolescents were satisfied with the care and treatment they received from their pediatrician (87%). Somewhat fewer adolescents were satisfied with the psychiatrist (76%), while just over half were satisfied with their FBT therapist (57%). When asked to rate the program as a whole, 89% of parents and 72% adolescents were satisfied with the care and treatment they received.

*“From the moment we entered we felt surrounded by expert help. We were guided through everything and even though recovery took a long time, it did happen and we are so thankful. The doctors we had were amazing, so helpful and understanding, but at the same time firm and very clear in their directions to us.”*—Parent

*“The unbound kindness and help from all the doctors and team members during my treatment. Whatever concerns or questions I had, they supported me and gave me solutions to work through them.”*—Adolescent

### Perceptions of Recovery

When asked about signs of recovery that were achieved during their time with the program, there was considerable variation (see **Table 5**). Most parents thought their child reached a healthy weight (82%) and achieved physical health (80%) during their time with the program. However, only 61% thought their child achieved good mental health. Around half the parents (53%) thought their child had recovered during this time. Similarly, while 72% of adolescents thought they achieved a healthy weight and 68% thought they had physically recovered, just 45% thought they had achieved good mental health; 56% thought they had recovered during their time with the program.

*“The outcome was stabilization of our daughter and that probably saved her life, but not complete recovery.”*—Parent

*“Even though my weight is normal, I don’t feel like I am psychologically free from this eating disorder.”*—Adolescent

**Table 5:** Perceptions of recovery.

	Parents (n = 145)			Adolescents (n = 85)		
*How much do you agree you [your child] was able to achieve each of the following at some point during your time with the program?*	n (%)	n (%)	n (%)	n (%)	n (%)	n (%)
*I [my child]…*	Disagree	Neutral	Agree	Disagree	Neutral	Agree
1. … reached a healthy weight	12 (8.8)	13 (9.5)	112 (81.8)	13 (15.9)	10 (12.2)	59 (72.0)
2. …achieved physical health	12 (8.8)	15 (11.0)	109 (80.1)	14 (17.1)	12 (14.6)	56 (68.3)
3. …achieved mental health/wellbeing	34 (24.8)	20 (14.6)	83 (60.6)	28 (34.1)	17 (20.7)	37 (45.1)
4. …recovered from their eating disorder	43 (31.4)	22 (16.1)	72 (52.6)	24 (29.3)	12 (14.6)	46 (56.1)

### Experience of Discharge Processes

With regard to discharge processes, 16% of parents and 29% of adolescents did not feel they were well prepared for the end of FBT and 15% of parents and 20% of adolescents did not feel they were well prepared for discharge from their pediatrician (see **Table 6**). Similarly, 18% of parents and 19% of adolescents reported that they did not receive appropriate referral to other services, and 21% of parents and 23% of adolescents reported that they did not receive enough information about adult services. Furthermore, while 76% of parents reported that they knew what to do if their child relapsed, just 47% of adolescents reported that they knew what to do if they relapsed.

**Table 6:** Experience of discharge.

	Parents (n = 145)			Adolescents (n = 85)		
	n (%)	n (%)	n (%)	n (%)	n (%)	n (%)
	Disagree	Neutral	Agree	Disagree	Neutral	Agree
***Thinking about the end of your [your child’s] care at the RCH Eating Disorders Program, how much do you agree with each of the following?***						
1. I was well prepared for the end of family based treatment	21 (16.0)	21 (16.0)	89 (67.9)	22 (28.6)	11 (14.3)	44 (57.1)
2. I was well prepared for discharge from seeing the medical doctor	18 (14.6)	15 (12.2)	90 (73.2)	15 (19.7)	11 (14.5)	50 (65.8)
3. I was provided with appropriate referrals to other services (parent only)	22 (17.5)	17 (13.5)	87 (69.0)	–	–	–
4. I was assisted to continue treatment at another service (adolescent only)	–	–	–	14 (18.9)	15 (20.3)	45 (60.8)
5. I [*We*] received enough information about adult services	24 (20.5)	24 (20.5)	69 (59.0)	17 (23.3)	23 (31.5)	33 (45.2)
6. I knew what to do if I [*my child*] relapsed	16 (12.2)	16 (12.2)	99 (75.6)	24 (30.4)	18 (22.8)	37 (46.8)

*“When the program finished we had nowhere to go. We were left to our own devices. There needs to be more care beyond FBT.”*—Parent

*“When I was shifted off to the [adult] hospital (as I was too old to continue treatment at the children’s hospital) I felt like everything changed. It wasn’t a smooth transition.”*—Adolescent

## Discussion

This study provides important insights into the experience of care of adolescents and their parents in a specialist eating disorder service providing FBT as its primary treatment modality. It is novel in its inclusion of a relatively large adolescent sample, the exploration of both treatment-specific and broader service aspects of care, and its locality within a country with a federally-funded healthcare system. Reassuringly, we found that, despite the challenges that treatment of restrictive eating disorders such as AN can present, families were generally very positive about their experience of care within a specialist adolescent FBT service. Consistent with previous research (16–19), parents tended to be more satisfied with the service than were adolescents, although on the whole, most adolescents also held the service in high regard. Nonetheless, several areas were identified where greater attention could improve the experience of adolescents and their parents and potentially improve engagement and outcomes of FBT.

Of note, many families reported a delay in accessing treatment. This is important given that shorter duration of symptoms is associated with more favorable prognosis (26). Many parents attributed delays to general practitioners not recognizing symptoms or the need for referral, or because families seeing other practitioners prior to referral, such as psychologists and dietitians who did not specialize in eating disorders. Several parents also reported delays in getting an appointment with the service. Although the wait time for an appointment at this service is generally only a few weeks, a triage system operates to prioritize patients who are very unwell which can mean that at busy times patients with less severe symptoms may have to wait longer for an appointment. Unfortunately, this can mean symptoms worsen before the adolescent can be assessed. These findings point to a need for greater education for clinicians in primary care, mental health, and dietetics, as well as a need for greater investment in specialist services to enable them to respond and provide care in a timely manner.

Once families were able to access the service, most had a very positive experience of the multidisciplinary intake assessment and felt it provided them with a good understanding of the diagnosis and need for treatment. Of importance, however, only around half of adolescents reported that they felt safe and comfortable to talk about their problems at this assessment. This may be understandable given that completing multiple assessments with different clinicians, usually in a single day, allows little time to build rapport with the adolescent. This may be exacerbated by the fact that many adolescents, and also some parents, felt that other causes for eating and weight problems were not considered and that other physical and mental health problems were neglected. An alternative approach used by some other services, is to spread intake assessments over several days or weeks. This may give additional time for clinicians to build rapport with the family and demonstrate that other issues are being given full consideration. However, the one-day intake assessment at this service has the benefit of providing a comprehensive evaluation in a relatively short period of time so that FBT can commence immediately. First appointments are typically within one week of assessment and any outstanding investigations are followed up by the team concurrently. Within this format, the assessing clinicians might better demonstrate a holistic approach by enquiring further about other problems at each stage of the assessment, being explicit about how differential diagnoses are evaluated, and checking in with the family that they are satisfied that other causes and issues have been given due consideration.

Moving into treatment, parents felt that they received appropriate education about eating disorders and most felt they received sufficient guidance in key areas of FBT such as meal support and prevention of compensatory behaviors. They were also very positive about the team members and thought their child’s health and wellbeing were monitored and responded to appropriately. Of importance, parents did not feel the team blamed them for their child’s illness. This is a key tenet of FBT which aims to alleviate parents’ feelings of guilt and empower them to help their child (4). In contrast to these very positive views, many parents wanted more information on evidence for treatment and medications, and many parents wanted greater guidance in supporting their child to cope with weight gain and how to manage their own distress during treatment. Previous research has similarly identified a need for carer support in eating disorder services (22, 23), which is especially relevant to FBT given the central role of parents. Since the survey was administered, a parent education and support group has been implemented at the current service (27). This group is attended by all parents in the first few weeks of treatment and provides information on topics including treatment evidence and how to support adolescents during treatment, as well as providing parental peer support. Other carer support approaches include the New Maudsley Method which is a treatment adjunct designed to reduce the stress and anxiety associated with caring for a person with an eating disorder (28). Finding ways to support parents during treatment could not only have benefits for parents’ wellbeing but may also promote engagement, more adept skills in managing and treating the illness and, in turn, improved outcomes for patients (29).

Like their parents, and possibly more so, adolescent wanted more information on evidence for treatment and medications, as well as prognosis. When and how best to provide this needs further consideration given that some adolescents expressed they were too overwhelmed early in treatment to absorb much information. In addition, adolescents were less positive about their interactions with the team than were parents. While the vast majority felt they were treated with respect and that their health and wellbeing were well monitored (i.e., 82–94%), notably fewer had confidence in the team’s ability to help them and felt that their concerns were listened to and acted on appropriately (64–67%). Some adolescents responded neutrally to these statements (i.e., neither agreeing nor disagreeing); however, other adolescents felt quite negative about the team. This was most apparent in ratings of clinicians, as a quarter of adolescents reported being dissatisfied with the care and treatment they received from their FBT therapist. It is perhaps unsurprising given that the therapist is central in driving behavioral changes which are difficult for someone with AN, many of whom are likely to be resistant to treatment (30). Indeed, previous research confirms that adolescents tend to report lower therapeutic alliance with FBT therapists than do parents (31). Building and maintaining therapeutic alliance with the adolescent while achieving the goals of FBT is a difficult balance; however, research suggests that greater alliance with the therapist predicts better outcomes for adolescents (32). Exploration of how therapists can achieve and maintain therapeutic alliance with adolescents in the context of FBT requires greater attention.

One area that could be explored with regard to therapeutic alliance was the experience of some adolescents that their concerns and distress were not listened to and acted on. From responses to the open-ended questions, this experience was frequently related to the focus on physical health (including weight gain) and behavioral symptoms which some adolescents perceived was neglectful of their mental health. A lack of individual therapy and attention to underlying issues are common themes in previous qualitative research on family therapies for AN (14) including FBT (16). This is also consistent with the findings of the current study regarding perceptions of recovery, in that most parents and adolescents reported that the adolescent achieved physical health but much fewer thought that good mental health was achieved. The urgent physical health complications of AN mean that restoring physical health through weight gain is prioritized in FBT for AN. However, ensuring mental health issues are addressed with the adolescent and their family during treatment is clearly of importance to the experience of care. Beyond FBT, consideration also needs to be given to what follow up might be needed for comorbid conditions and by whom. Given the focus of specialist eating disorder services and their limited resources, these may best be managed by another service; however, greater communication with families around this may be indicated.

When FBT comes to an end and discharge and/or transfer to other services is needed, this can be a challenging time, regardless of families’ experience to date. Although most parents and adolescent were satisfied with their discharge experience, several expressed that they did not feel well prepared for the end of treatment or did not receive appropriate referrals to other services or information about adult services. Importantly, less than half of adolescents reported that they knew what to do if they relapsed. A transition service was in operation during this study which aimed to support adolescents being transferred to the adult hospital; however, this was not available to all families (e.g., younger adolescents, those not in the geographical catchment). Other discharge support efforts have since been implemented including three “booster sessions” which are provided during the 6 months following FBT to monitor symptoms, reduce risk of relapse, and provide a less abrupt cessation of treatment with the FBT clinician. While these efforts may go some way to improving the experience of discharge, more research into how services can support families during this time is needed. This may ultimately require greater investment in resources to allow specialist services to provide longer or more individualized approaches.

This study has several strengths which make the findings of importance to the provision of services to families of adolescents with restrictive eating disorders. To our knowledge, this is the largest survey of consumer views of a specialist FBT service to have been conducted for adolescents with AN and other restrictive eating disorders. Previous large surveys have been with current or former adult patients and carers with experience of a range of diagnoses and services, while studies of family therapies have been mostly small qualitative studies, or have focused on the components of FBT rather than the broader service in which FBT is delivered. In addition, by designing a survey for our specific service, we were able to capture parents’ and adolescents’ experience of care in a more nuanced manner than a standardized service satisfaction scale would allow. However, this type of service-specific measure does have limitations. Psychometric aspects such as reliability and validity cannot be demonstrated, and there may be bias inherent in the selection and design of survey items. Importantly, the specificity of the survey might mean it cannot be readily used in other service settings. Another feature of the survey, the open-ended questions at the end of the survey, provided important context to supplement the quantitative findings. The responses were not sufficient for a rigorous qualitative analysis; however, they gave a voice to the parents and adolescents who participated beyond what can be expressed in rating scales alone. Future studies might benefit from applying a mixed-methods approach which more fully explores families’ experience of care in eating disorder services.

The moderate response rate could be considered a limitation of the study; however, that over two-thirds of eligible families were represented is an achievement given the nature of the research. Furthermore, only around half the sample reported achieving full recovery during treatment, suggesting that participation was not necessarily skewed toward those who had a more positive experience. That said, fathers were not well represented, which was in part due to mothers declining to pass surveys on to fathers to complete. Fathers are expected to be fully involved in FBT, and their attendance at treatment sessions is associated with better patient outcomes (33). Engaging them in a survey such as this could be important for uncovering unique factors that contribute to the engagement of fathers. Likewise, including greater numbers of participants across different diagnostic classifications could be informative. There were not sufficient numbers in the current study for subgroup comparisons; however, larger studies could explore whether individuals with diagnoses such as AN, atypical AN, and ARFID have different experiences of care related to variations in clinical features and treatment approach.

An important limitation was that for some families the invitation to participate was up to four years since they received treatment at the service. This may have reduced their ability to recall details of their treatment, or their experiences since treatment may have influenced how they retrospectively perceived the quality of care. The delay may also have impacted on response rates due to waning motivation to participate or changes in contact details. Prospective collection of data on experience of care (e.g., during treatment or at point of discharge) would be the preferred design but was not possible due to constraints on time and funding. However, services should consider including prospective assessment of experience of care in their processes. The development of standardized measures would be especially beneficial in this regard to enable benchmarking across services which can subsequently inform service development including distribution of funding.

Overall, the study provided many insights into the experience of care of both parents and adolescents at a specialist multidisciplinary eating disorder service proving FBT as its primary model of care. As the preferred treatment for adolescent AN, it was reassuring that most parents and adolescents were positive about their experience. In particular, they were positive about the multidisciplinary assessment process, education about eating disorders, monitoring of adolescents’ physical and mental health, expertise of the team, and the care they received from their treating team. Nonetheless, there were some important indicators of areas that could be improved including treatment delays, carer support, therapeutic alliance, and preparation for discharge. This study provides direction for aspects of care that services could focus on to improve the experience of adolescents and their parents. Further research is needed to better understand the extent to which the experience of FBT and the broader treatment setting relate to outcomes (including relapse) and to explore the most effective options for addressing areas of concern. Subsequent investment in resources for specialist services will be needed to implement strategies that optimize treatment experience and outcomes. There may also be benefit in incorporating standardized repeat surveys across services to allow for benchmarking and evaluation of the effectiveness of strategies to enhance families’ experience of care.

## Data Availability Statement

The datasets generated for this study are available on request to the corresponding author.

## Ethics Statement

The studies involving human participants were reviewed and approved by Royal Children’s Hospital Human Research Ethics Committee, Melbourne, Australia. Written informed consent from the participants’ legal guardian/next of kin was not required to participate in this study in accordance with the national legislation and the institutional requirements.

## Author Contributions

SS conceptualized the study. EH supervised the project, designed and facilitated the study, supervised and undertook the analyses, interpreted the results, drafted the manuscript, and approved the final version. SP and AB contributed to obtaining and cleaning the data, initial data analysis, interpreting the results, and writing the manuscript. MY, MT, and SS supervised the project, designed and facilitated the study, and contributed to interpreting the results and editing the manuscript.

## Funding

This work was supported by the Baker Foundation (Australia) and the University of Melbourne. The Murdoch Children’s Research Institute is supported by the Victorian Government’s Operational Infrastructure Support Program.

## Conflict of Interest

The authors declare that the research was conducted in the absence of any commercial or financial relationships that could be construed as a potential conflict of interest.

The reviewer MK declared a past co-authorship with one of the authors, EH, to the handling editor.
